# C3 deficiency promotes pulmonary inflammation in AT1R-induced mouse model for systemic sclerosis

**DOI:** 10.3389/fimmu.2024.1491324

**Published:** 2024-12-16

**Authors:** Junping Yin, Admar Verschoor, Xiaoyang Yue, Torsten Goldmann, Harald Heidecke, Gabriela Riemekasten, Frank Petersen, Xinhua Yu

**Affiliations:** ^1^ Priority Area Chronic Lung Diseases, Research Center Borstel - Leibniz Lung Center, Members of the German Center for Lung Research (DZL), Borstel, Germany; ^2^ Department of Otorhinolaryngology, Technische Universität München and Klinikum Rechts der Isar, Munich, Germany; ^3^ Department of Dermatology, University Clinic of Schleswig Holstein, University of Lübeck, Lübeck, Germany; ^4^ College of Basic Medical Science, Key Laboratory of Basic Research on Regional Diseases, Education Department of Guangxi Zhuang Autonomous Region, Guangxi Medical University, Nanning, China; ^5^ Histology, Research Center Borstel - Leibniz Lung Center, Members of the German Center for Lung Research (DZL), Borstel, Germany; ^6^ CellTrend GmbH, Im Biotechnologiepark, Luckenwalde, Germany; ^7^ Department of Rheumatology and Clinical Immunology, University Clinic of Schleswig Holstein, University of Lübeck, Lübeck, Germany

**Keywords:** systemic sclerosis, inflammation, mouse model, complement, pulmonary inflammation

## Abstract

**Introduction:**

Autoantibody-mediated complement activation plays an essential role in a variety of autoimmune disorders. However, the role of complement in systemic sclerosis (SSc) remains largely unknown. In this study, we aimed to determine the role of complement C3 in the development of a recently described SSc mouse model based on autoimmunity to angiotensin II receptor type 1 (AT1R).

**Methods:**

Mice were immunized with cell membrane extract isolated from Chinese hamster ovary (CHO) cells overexpressing AT1R or non-transfected CHO cells as a control. Peripheral blood, dorsal skin and the lung were then collected to evlauate disease characteristics. Apoptotic cells in the lung of mice were detected using the DeadEnd™ Fluorometric TUNEL System.

**Results:**

Our results showed that experimental SSc in this model was featured by the deposition of IgG, but not of complement C3, in the lung. After immunization with AT1R, C3-deficient mice developed more severe pulmonary inflammations than wild type controls, whereas skin inflammation and fibrosis were not different as well as the anti-AT1R ab levels. Further, C3-deficient mice showed an increased rate of pulmonary cell apoptosis as compared to controls. The apoptosis rate correlated with the corresponding degree of lung inflammation.

**Discussion:**

Taken together, our findings suggest an anti-apoptotic and anti-inflammatory role of complement C3 in pulmonary autoimmune inflammation.

## Introduction

Systemic sclerosis (SSc) is an autoimmune connective tissue disease with multiple organ involvements and heterogeneous clinical manifestations (1). It is characterized by autoimmunity, inflammation, vasculopathy and fibrosis in skin and visceral organs (2). Although the disease prevalence is relatively low compared to other autoimmune disorders, SSc is featured by a high mortality rate mainly due to disease manifestations in the lung (3). Multiple autoantibodies (abs) are present in sera of patients with SSc. Some of them have been suggested to play a role in the development of SSc (4, 5). For example, high levels of abs against angiotensin II receptor type 1 (AT1R) are present in the serum of SSc patient. They are associated with fibrotic and vascular SSc manifestations and predict disease-related mortality (6). *In vitro*, anti-AT1R abs are able to induce the expression of chemokines and adhesion molecules in endothelial cells and collagen type-1 in fibroblasts (6–8), suggesting a potentially pathogenic role of these abs. Recently, we have verified this notion by immunizing mice with membrane extract from Chinese Hamster Ovary (CHO) cells overexpressing AT1R. After the immunization, C57BL/6J mice generated receptor-activating functional abs against AT1R as well as fibrosis and inflammation in the skin and lung, resembling multiple SSc features (9).

Autoantibodies are capable of inducing pathological alteration via multiple pathways, which generally can be categorized into 2 groups: Fc-dependent and Fc-independent (10). The Fc-dependent pathways stimulate immune cells by their Fc receptors and activate the complement system via immune complexes. Here, complement activation by autoantibody-antigen complexes lead to the deposition of a sublytic membrane attack complex and release of chemotactic and inflammatory factor such as C5a. This essential mechanism of autoantibody-mediated pathology was shown in various autoimmune diseases including myasthenia gravis (11), pemphigoid diseases (12) and rheumatic disorders (13). In contrast, the Fc-independent pathways are mediated solely by antigen binding domain of abs (10). It has been reported that SSc is featured by decreased circulating levels of C3 and C4 (14, 15). Moreover, biomarkers of complement system activation including C3d and C4d are increased in SSc patients as compared to healthy controls. The levels of those biomarkers are associated with disease manifestations such as scleroderma renal crisis (16, 17). Despite these clinical findings suggest that complement activation is associated with the development of SSc or at least of some SSc manifestations, the contribution of complement to the pathogenesis of SSc has not been investigated (18). Animal models are excellent research tool for investigating the pathogenesis of human disorders (19). They have been extensively applied to study the role of complement in experimental models for various autoimmune diseases, but not SSc (18, 20). Thus, although the complement system is thought to contribute to the pathogenesis of various autoimmune rheumatic diseases, its role in the development of SSc remains largely unknown (21).

In this study, we aimed to determine the role of complement system in SSc using the AT1R-inducde mouse model for SSc. The complement system, a central component of innate immunity, is essential for defense against pathogens and maintaining host homeostasis (22). It can be activated through three distinct pathways—classical, alternative, and lectin—all of which require C3 for proper function (22). Therefore, we determined the role of C3 in the mouse model for SSc using C3-deficient mice. Our results revealed an unexpected anti-inflammatory role of C3 in pulmonary inflammation.

## Methods

### Mice

Seven-week old female wild type C57BL/6J mice were purchased from the Jackson Laboratory (Bar Harbor, ME, US). Complement C3-deficient mice (B6.129S4-C3^tm1Crr^/J, C3 KO) were obtained from University of Lübeck, Lübeck, Germany. All mice were housed under specific pathogen free conditions with 12-hour light/darkness cycles at the animal facility of Research Center Borstel, Germany. All animal experiments were reviewed and approved by the Animal Research Ethics Board of the Ministry of Energy Change, Agriculture, Environment, Nature, and Digitalization, Kiel, Germany (Number 104-8/17).

### Immunization with membrane extract

Cell membrane extract (ME) isolated from Chinese Hamster Ovary (CHO) cells overexpressing AT1R or non-transfected CHO cells were kindly provided by CellTrend (Luckenwalde, Berlin, Germany). Eight to ten weeks old female mice were immunized with 0.2 mg of control or AT1R ME in 50 µl of PBS emulsified with complete Freund adjuvant (CFA, Sigma-Aldrich, USA) by subcutaneous injection into the footpad as described previously (9). Three weeks after the immunization, the mice were boosted with the same amount of antigen emulsified with incomplete Freund adjuvant (IFA, Sigma-Aldrich, USA). Nine weeks after the first immunization, peripheral blood, dorsal skin and the lung were harvested for the evaluation of disease characteristics.

### Detection of deposition of IgG and C3

The deposition of IgG and C3 in the murine lung were determined by immunofluorescence staining. Briefly, the lung samples were collected from mice and cryosection was performed at thickness of 3 um, after fixation in pre-cold acetone and blocking in blocking buffer (Carl Roth, Germany), IgG binding was detected using DyLight 649-labeled goat anti-mouse IgG antibody (polyclonal, BioLegend, USA), complement 3 was detected with rat anti-mouse C3 antibody (RMC11H9, Cedarlane, Canada) and Alexa488 labled-goat anti-rat IgG antibody (polyclonal, Initrogen, USA). Coverslips were mounted with Gold mounting agent (Thermo Fisher, USA) that contains 4`, 6-diamidino-2-phenylindole (DAPI) for nuclear staining, the fluorescence was evaluated by confocal microscopy at a magnification indicated in the legends to figures (Leica SP5, Germany). The intensity of the fluorescent signals was analyzed and quantified using Image J software (version 1.53t, USA).

### Quantification of anti-AT1R antibodies

The level of anti-AT1R autoantibodies in the mouse serum samples were detected by using enzyme-linked immunosorbent assay (ELISA) as previously described (9). Levels of anti-AT1R antibodies were defined as the dilution at which the OD value reached the half of maximal OD values of the curve.

### HE staining

H&E staining was performed to determine the inflammation in tissues. Paraffin-embedded sections from mouse skin and lung were deparaffinized in xylene and rehydrated in ethanol series with decreasing concentrations, then stained with hematoxylin and eosin solutions. Thereafter, the stained sections were dehydrated and then mounted with mount medium. Severity of pulmonary and skin inflammation was quantified by the size and number of infiltrates in the lung and skin, and scored in a double-blinded fashion as described previously (9).

### Masson’s Trichrome staining

To evaluate tissue fibrosis, paraffin-embedded tissue sections of mouse skin and lung were stained with Masson’s Trichrome staining kit (Sigma-Aldrich, USA). Tissue sections were deparaffinized in xylene and rehydrated in ethanol series with decreasing concentrations. Rehydrated sections were fixed in Bouin’s solution at RT overnight, counterstained with Hematoxylin Gill II solution, washed and incubated with Biebrich scarlet-Acid Fuchsin, and subjected to phosphotungstic/phosphomolybdic acid solution. Phosphoric acid treated sections were then incubated with aniline blue solution, differentiated in 1% acetic acid, rinsed with deionized water, dehydrated in ethanol, and finally mounted with mounting medium. Skin thickness was measured as the thickness of the blue collagen layer.

### Measurement of skin collagen content

The collagen content of the skin was determined by using the Sircol collagen detection kit (Biocolor, UK) according to the manufacture’s instruction. Briefly, punches of skin tissues (9 mm^2^) were cut into pieces and digested in 0.5 M acetic acid solution containing porcine pepsin (Sigma, USA) at 4°C overnight on the shaker. In the next day, supernatant was collected and was mixed with Sircol dye solution and incubated on shaker. Afterwards, samples were centrifuged and pellets were washed with ice cold acid salt wash reagent to remove unbound dyes. Dye bound collagen pellets were dissolved in Alkali buffer was added to each sample to release collagen. To record the OD values, samples were transferred into a plate and measured on TECAN microplate reader at a wavelength of 550 nm. Amount of collagen in samples were calculated according to the standard curve generated with gradient diluted collagen.

### Immunohistochemistry

Immunohistochemistry staining was conducted on murine tissue sections to assess the cellularity of infiltrated cells. Lung paraffin-embedded sections underwent deparaffinization in xylene followed by rehydration in a gradient of ethanol. Subsequently, antigen retrieval was achieved by microwave heating with 1× citrate buffer (pH=6) (ZytoMed, BE, Germany, Cat#ZUC028-500), and sections were blocked using 3% H_2_O_2_. They were then incubated with specific primary antibodies for 1 hour at room temperature, including rabbit anti-CD20 (Invitrogen, MA, USA, Cat# PA5-16701, 1:500), rabbit anti-CD3 (ZytoMed, Cat# RBK024-05, 1:100), or rat anti-mouse neutrophil antibodies (Cedarlane, ON, Canada, Cat# CL8993AP, 1:800). Following washing steps, the sections were treated with ImmPRESS-HRP goat anti-rat IgG polymer kit (VECTOR, Cat# MP-7404-50) or ZytoChem Plus-HRP polymer kit (ZytoMed, Cat#POLHRP-100), followed by incubation with 3-Amino-9-ethylcarbazole (AEC) (ZytoMed, Cat#ZUC054-200) to visualize immunoreactivity. Finally, counterstaining with hematoxylin was performed, and bright-field microscopy was used to capture images.

### Assessment of cell apoptosis in the lung tissue

The apoptotic cells in the lung of mice were detected with DeadEnd™ Fluorometric TUNEL System (Promega, USA) according to the manufacturer recommended procedures. Briefly, the deparaffinize and rehydrated lung sections were permeabilized with 0.2% Triton X-100 and equilibrated in equilibration buffer. Immediately before the reaction, the TUNEL mixture was freshly prepared and added to the sections. After incubation with rTdT incubation buffer, the reactions were stopped by SSC buffer. Sections were finally mounted with mounting medium containing DAPI for counterstaining. To quantify the level of apoptotic cells, six to ten random confocal images were recorded and the number of apoptotic cells and total cells were measured, and the rate of apoptotic cells were calculated as number of apoptotic cells divided by total number of cells.

### Statistics

All data was analyzed by using Graphpad Prism software (version: prism 10.2). Statistical differences of quantitative data between two groups were determined by two-tail unpaired Student’s t test. Two-way ANOVA was used to determine the statistical significance among multiple groups with two category variables. Significant differences between qualitative datasets were determined by Fisher-exact test. A *p* value below 0.05 was considered as statistically significant.

## Results

### AT1R immunization induces IgG deposition but not C3 deposition in murine lung

**Figure 1:**
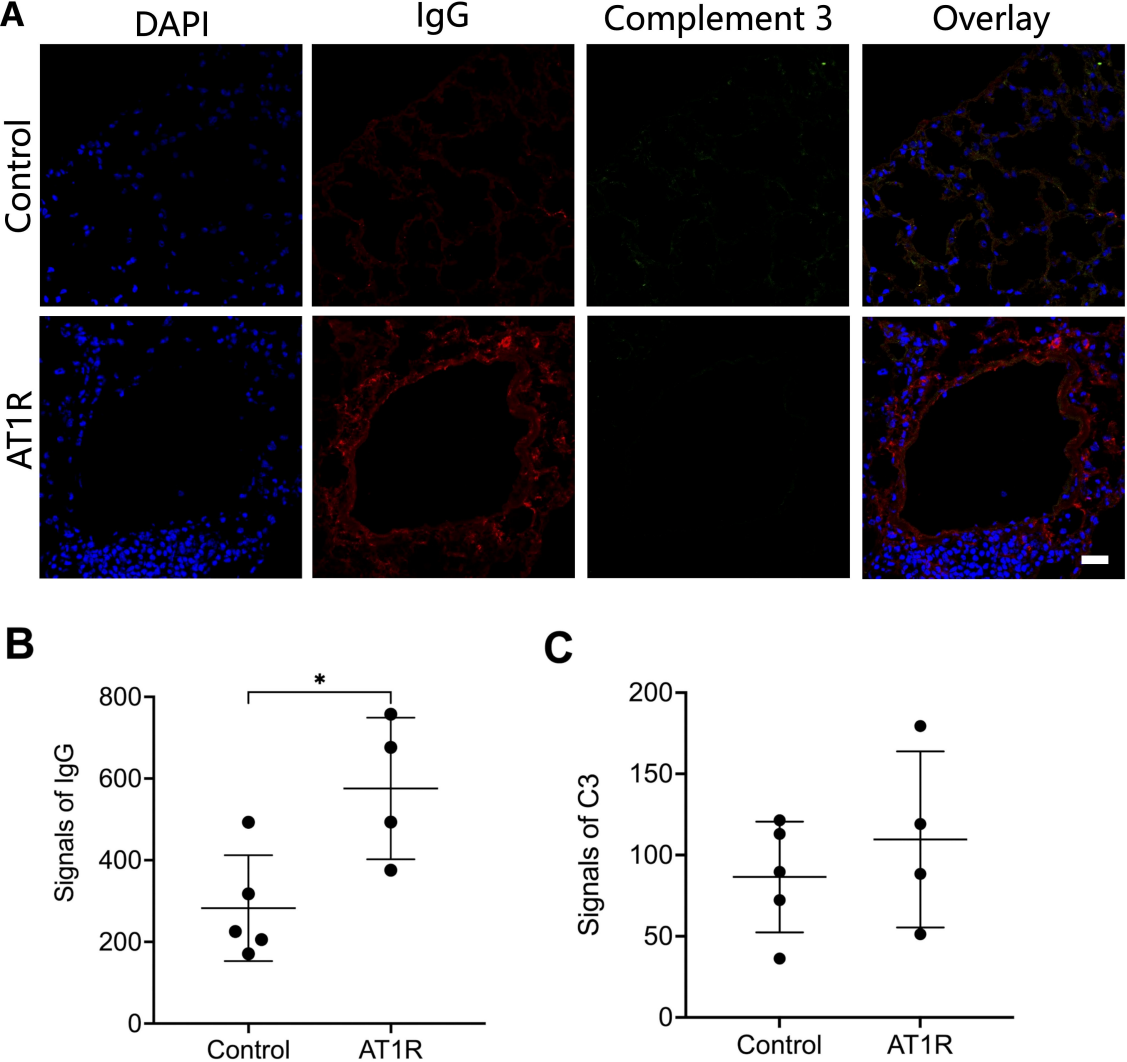
Deposition of IgG but not complement 3 in lung tissue of hAT1R-immunized mice. **(A)**. Cryosections of lung tissue from hAT1R-immunized mice or control mice were incubated with DyLight 649-labeled goat anti-mouse IgG antibody to detect IgG deposition (red), complement 3 was stained with rat anti-mouse C3 antibody and Alexa488 labeled-goat anti-rat IgG antibody (green), nucleus was stained with DAPI (blue). The representative micrographs were shown (630x, scale bar= 25mm). **(B, C)**. The quantitative analysis of the fluorescent signals of IgG and complement 3 was performed with pictures of representative fields of each group (n=4~5) using ImageJ software. The results are presented as the mean ± SD, and the statistical analysis was performed by using Student’s t-test (*: p<0.05).

To investigate the role of complement activation in the AT1R-induced mouse model for SSc, we first determined the deposition of IgG and complement C3 in the lung. Direct Immunofluorescence staining revealed that mice immunized with AT1R showed significantly higher levels of IgG deposition in the lung than mice immunized with control ME (**Figures 1A, B**). By contrast, no significant difference in C3 deposition was observed between AT1R-immunized mice and controls suggesting the lack of IgG-mediated complement activation (**Figures 1C**). In contrast, C3 deposition was effectively detected in a mouse model of epidermolysis bullosa acquisita (**Supplementary Figure 1**), where complement activation is a crucial step in disease progression (23).

### C3 deficiency promotes AT1R-induced pulmonary inflammation

**Figure 2:**
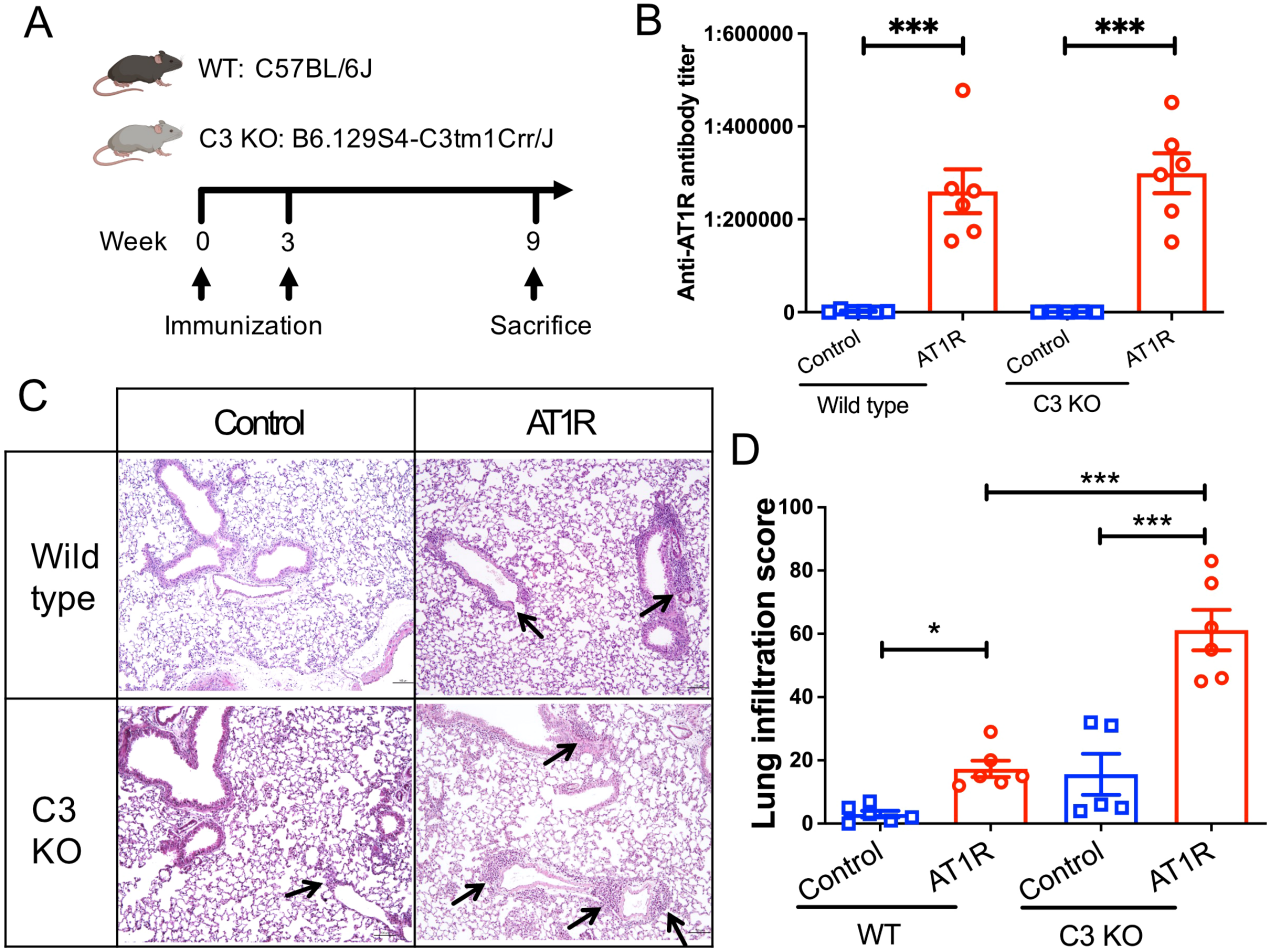
C3 deficiency promotes the AT1R immunization-induced lung inflammation. **(A)**. Schematic diagram of immunization protocol and groups. Mice were immunized at week 0 with membrane extract (AT1R ME or control ME) and boosted 3 weeks later. Nine weeks after the first immunization, mice were sacrificed, and blood and tissue samples were collected for evaluation. Five to six mice were analysed in each group. **(B)**. Levels of anti-AT1R IgG were determined in sera by ELISA. **(C)**. Representative micrographs of H&E-stained lung sections of control ME- or AT1R ME-immunized mice. *Black arrows indicate inflammatory* cell *infiltration* in the lung. Scale bar = 100 µm. **(D)**. Quantitative analysis of pulmonary inflammation in mice. Statistical analysis was performed by using two-way ANOVA test. * p<0.05, ***, p<0.001.

To better examine the role of complement C3 in this mouse model, C3 deficient mice and wild type (WT) control mice were immunized with AT1R ME or control ME. Nine weeks after the first immunization, blood, lung and skin were collected for further evaluation (**Figure 2A**). As shown in **Figure 2B**, immunizations with AT1R induced comparable levels of anti-AT1R IgG in C3 deficient and WT mice, suggesting that C3 deficiency does not affect the production of abs against AT1R.

Histological examination of lung tissue showed that AT1R immunization induced pulmonary inflammation in both C3 deficient mice and WT controls. Notably, C3-deficient mice developed a more severe AT1R-induced inflammation in the lung than WT mice (**Figures 2C, D**, *p*<0.001). Immunohistochemistry analysis revealed that lung inflammation primarily consisted of T cells, with neutrophils and B cells also present, and the composition of lung infiltrates was comparable between AT1R-immunized C3 deficient and WT mice (**Supplementary Figure 2**). In addition, a trend of increased pulmonary inflammation, although not significant, in C3-deficient mice as compared to WT mice was also observed after immunization with control ME.

### C3 deficiency is dispensable for AT1R-induced disease manifestation in the skin

**Figure 3:**
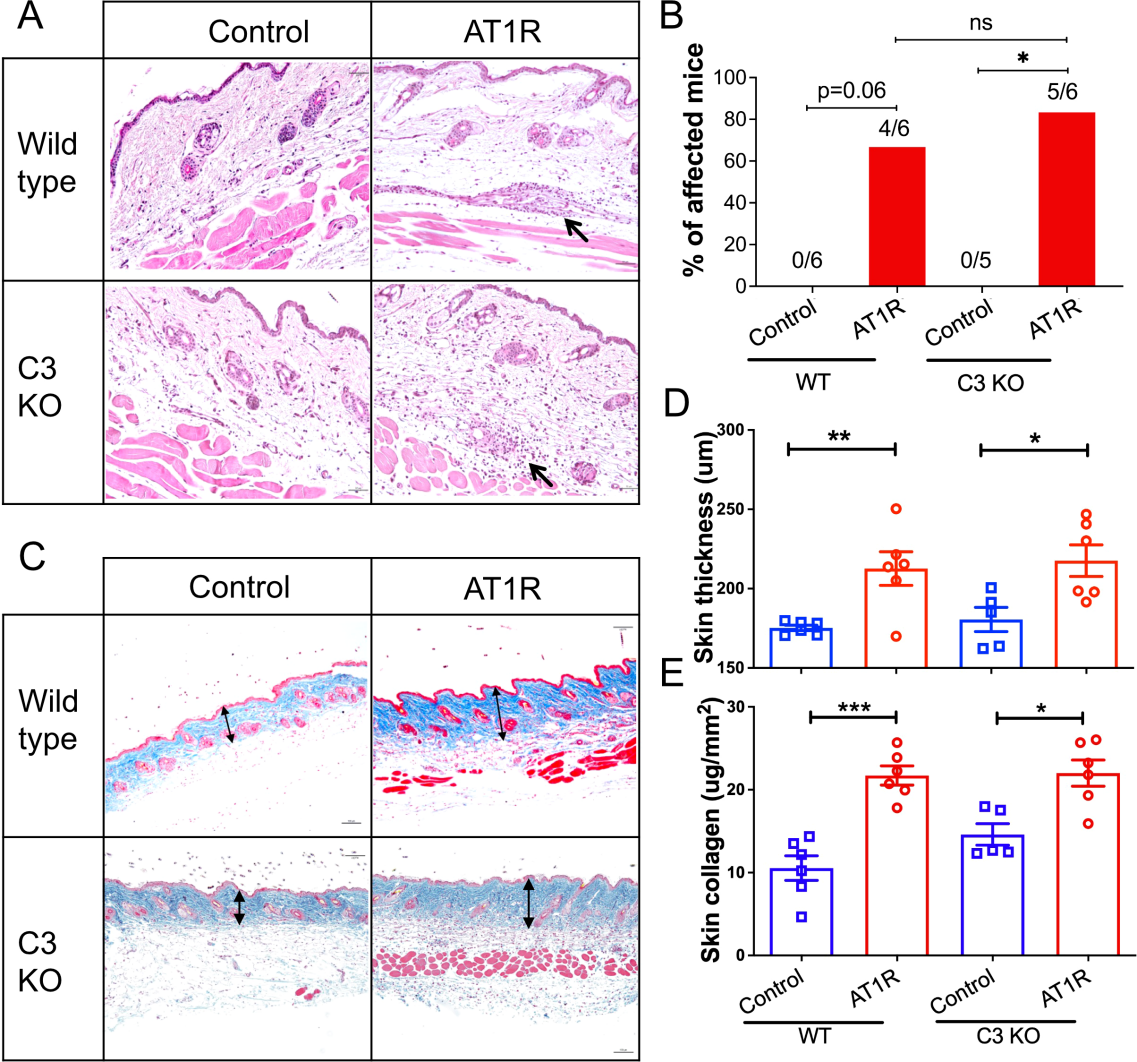
C3 deficiency does not affect skin inflammation. Wild type and C3-deficient (C3 KO) mice were immunized with control ME or AT1R ME. **(A)**. Representative micrographs of H&E-stained skin sections of control ME- or AT1R ME-immunized mice. *Black arrows indicate inflammatory* cell *infiltration* around blood vessels. Scale bar = 50 µm. **(B)**. Incidence of skin inflammation in mice. The numbers of mice with skin inflammation/all mice are presented on the top of corresponding bars. **(C)**. Representative micrographs of Masson’s Trichrome stained skin sections of control ME- or AT1R ME-immunized mice. Double-headed arrows indicate the collagen layer of the skin. Scale bar = 100 µm. **(D)**. Quantitative analysis of skin thickness. Thickness of the skin was defined as the thickness of the collagen layer stained in blue from the Masson’s Trichrome staining. **(E)**. Quantitative analysis of collagen content Collagen content was determined using Sircol collagen detection kit and expressed as μg per mm^2^ of the skin. Statistical analysis was performed by using two-way ANOVA test. *, p<0.05, **, p<0.01 ***, p<0.001.

We next examined the histopathological changes in the skin, another tissue involved in this experimental model (9). AT1R immunization induced comparable incidence of skin inflammation between WT controls and C3 deficient mice (**Figures 3A, B**). Furthermore, both skin thickness and collagen content were not significantly different between C3-deficient mice and WT controls immunized with AT1R (**Figures 3C–E**), indicating that C3 is dispensable for disease manifestations in the skin and indicate organ-specificity of the protective C3 effects for the lung.

### C3-deficiency promotes pulmonary cell apoptosis

**Figure 4:**
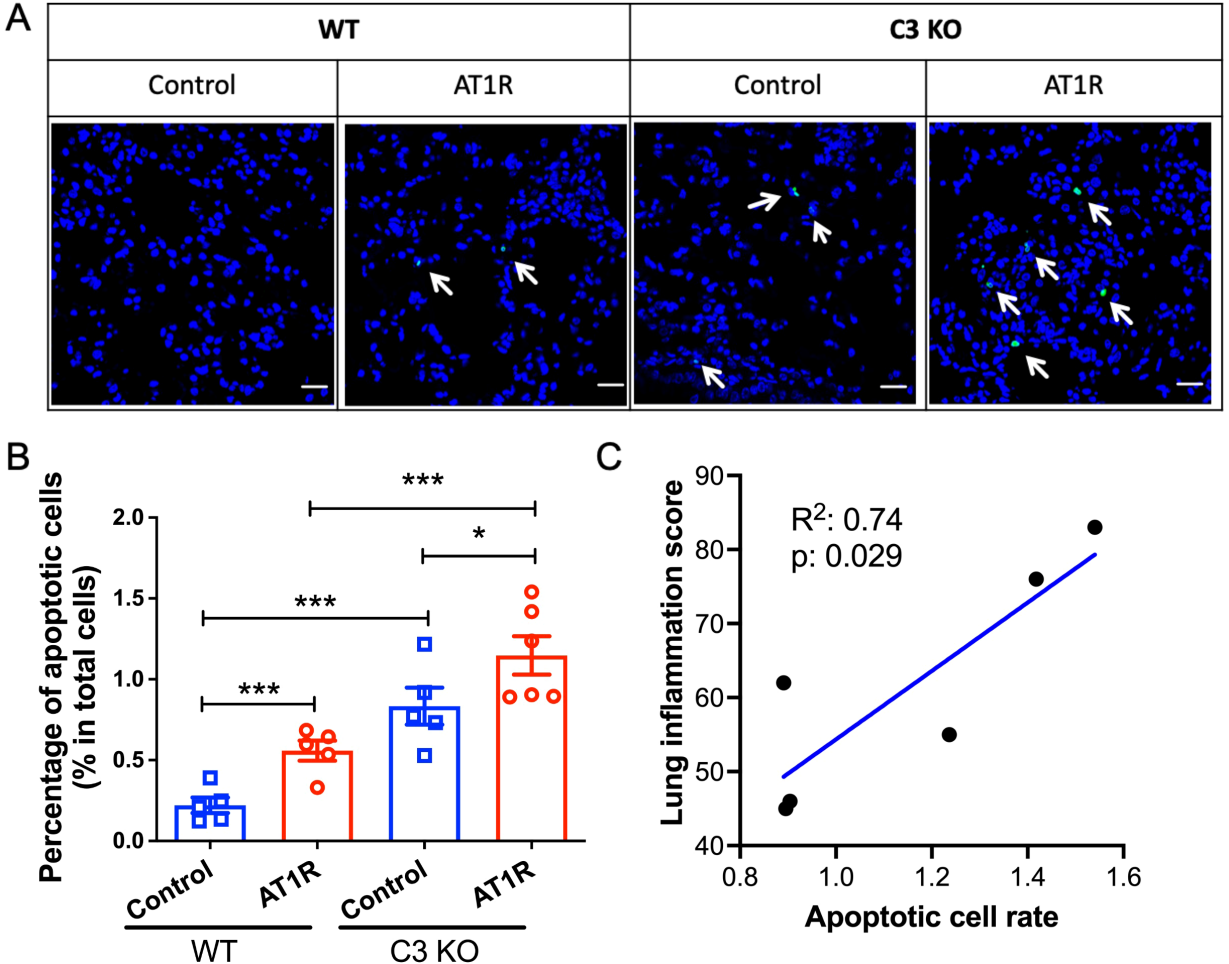
Increased apoptosis of lung epithelial cells in C3-deficient mice. **(A)**. Representative micrographs of apoptotic cells in lung cryosections from WT or C3 deficient mice visualized by TUNEL assay. Positive apoptotic cells are labelled in green fluorescence. Scale bar= 25mm. **(B)**. Quantitative analysis of apoptotic cells as determined in the TUNEL assay. The percentage of apoptotic cells in the total cells were presented as mean ± SD. Statistical analysis was performed by using two-way ANOVA test. *, p<0.05, ***, p<0.001. **(C)**. The correlation of severity of lung inflammation and apoptotic cell rate from the AT1R immunized C3 deficient mice. Blue line shows the simple linear regression line. R square and p values were presented in the plot.

In the next step we aimed to explore the mechanism underlying the effect C3 on pulmonary inflammation. Since it has been reported that C3 plays a protective role in apoptosis human airway epithelial cells and β-cells (24, 25), a common trigger of inflammation, we determined apoptosis of pulmonary cells in immunized mice. Compared to WT controls, C3-deficient mice showed a significantly increased pulmonary cell apoptosis rate after AT1R immunization (*p*<0.01) (**Figures 4A, B**). However, increased pulmonary cell apoptosis rate in C3-deficient mice as compared to corresponding WT controls was also observed in animals which received control ME (*p*<0.01). Finally, we evaluated the relationship between cell apoptosis and pulmonary inflammation. As shown in **Figure 4C**, a strong correlation was observed between pulmonary cell apoptosis rate and pulmonary inflammation in AT1R-immunized C3-deficient mice (R^2^ = 0.74, p=0.029), while such correlation was much weaker in the other three groups (data not shown).

## Discussion

Clinical studies have shown that SSc is featured by hypocomplementaemia and complement activation (14–17), implicating a role of complement system in the development of the disease which warrants further investigation. To our knowledge, this is the first study to examine the role of complement system in animal models for SSc.

In the present study, C3-deficient mice generated anti-AT1R autoantibodies and developed inflammation in the lung and skin as well as skin fibrosis. Moreover, IgG deposition, but not C3 deposition, was observed in the lung of AT1R-immunized wild type mice. Therefore, our results demonstrate that complement system is a dispensable factor for the disease development in the AT1R-induced mouse model. This notion is in line with the concept that anti-AT1R antibodies are functional autoantibodies which mediate disease pathology in a Fc-independent manner by a specific binding and stimulation of the AT1R (6, 9).

Previous studies have shown that C3 deficiency can impair the generation of antibodies against various pathogens and that C3 fragments such as C3b and C3d may act as a molecular adjuvant (26–30), suggesting a role for C3 in modulating humoral immunity. Interestingly, in our study, no significant difference in the antibody response against AT1R was observed between C3-deficient mice and wild-type controls. This discrepancy may be attributed to the strong adjuvant effect of CFA, which could potentially compensate for the absence of C3. This notion is supported by the observation that CFA indeed raises antibody responses similar to C3b as an adjuvant (30). Thus, the impact of C3 deficiency on humoral responses may be context-dependent.

Importantly, C3-deficient mice developed a more severe AT1R-induced pulmonary inflammation than wild type controls, while such difference was not observed in the disease manifestations in the skin. This finding suggests that C3 plays an anti-inflammatory and protective contribution specifically in the development of pulmonary inflammation. This finding suggests that enhancing C3 activity could be a potential therapeutic strategy for managing pulmonary manifestations in SSc. However, this clinical implication requires further evidence. Specifically, it is necessary to determine whether increasing C3 activity can inhibit lung inflammation in experimental models of SSc. Additionally, clinical data linking enhanced C3 activity with improved lung function are needed

It is noteworthy that C3a, typically considered a proinflammatory mediator, may also exhibit anti-inflammatory properties (31), supporting an anti-inflammatory role for C3. However, since C3 deposition was not observed in the lungs of this model, it is unlikely that the anti-inflammatory effect of C3 in this context is due to C3a. We propose instead that intracellular C3 may play an anti-inflammatory role by regulating pulmonary cell death, a mechanism suggested by previous studies (25, 32). In 2019, Kulkarni and colleagues reported that airway epithelial cells store intracellular C3 through biosynthesis and/or uptake of exogenous C3 during inflammation (25). Recently, Kulak et al. reported that intracellular C3 protects beta-cells from IL-1β-induced cytotoxicity via interaction with Fyn-related kinase (32). In line with this notion, recently Sahu et al. reported that complement C3 protects lung epithelial cells from bacteria induced lung inflammation and injury and such protection is through Factor B rather than C3a receptor (33).

One major limitation of the present study is that it employs only a single experimental model of SSc. Given the heterogeneous nature of SSc, each model represents only specific aspects of its pathogenesis. Therefore, the findings of this study should be interpreted with caution and not overgeneralized.

In conclusion, the present study showed that C3-deficient mice exhibited more severe lung inflammation and increased pulmonary cell apoptosis in a mouse model for SSc. These findings reveal an anti-inflammatory and anti-apoptotic effect of C3 in the lung. The unexpected anti-inflammatory role of C3 caries clinical implications, warranting further investigation.

## Data Availability

The original contributions presented in the study are included in the article/**Supplementary Material**. Further inquiries can be directed to the corresponding authors.
