# Supplementary material for: C3 deficiency promotes pulmonary inflammation in AT1R-induced mouse model for systemic sclerosis
Source: Front Immunol. 2024 Dec 16;15:1491324. doi: 10.3389/fimmu.2024.1491324 (PMC11683138; doi:10.3389/fimmu.2024.1491324)
Supplement: Supplementary file 1 [file DataSheet1.docx]

**Supplementary information**


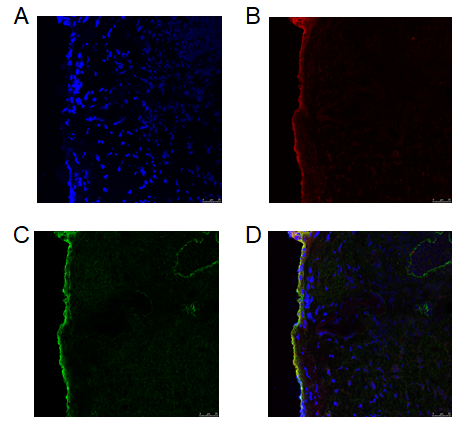


**Figure S1. Positive Control for Detection of Complement C3 Deposition in Murine Tissue**. Since complement C3 deposition is a critical step in disease progression within the passive mouse model of epidermolysis bullosa acquisita (EBA) (J Clin Invest. 2005 Apr;115(4):870-8), cryosections of skin tissue from mice in the EBA model were utilized as a positive control for the analysis. In this model, C57BL/6 mice were injected subcutaneously with rabbit anti-mCOL7 antibodies on days 0, 2, 4, 6, 8, and 10. On day 12, the mice were sacrificed, and skin tissue was collected to prepare cryosections. For detection, cryosections of murine skin were incubated with a DyLight 649-labeled goat anti-mouse IgG antibody to visualize IgG deposition (red, panel B). Complement C3 was stained using a rat anti-mouse C3 antibody, followed by an Alexa488-labeled goat anti-rat IgG antibody (green, panel C). Nuclear staining was achieved with DAPI (blue, panel A), and panel D shows the overlay of all stains.


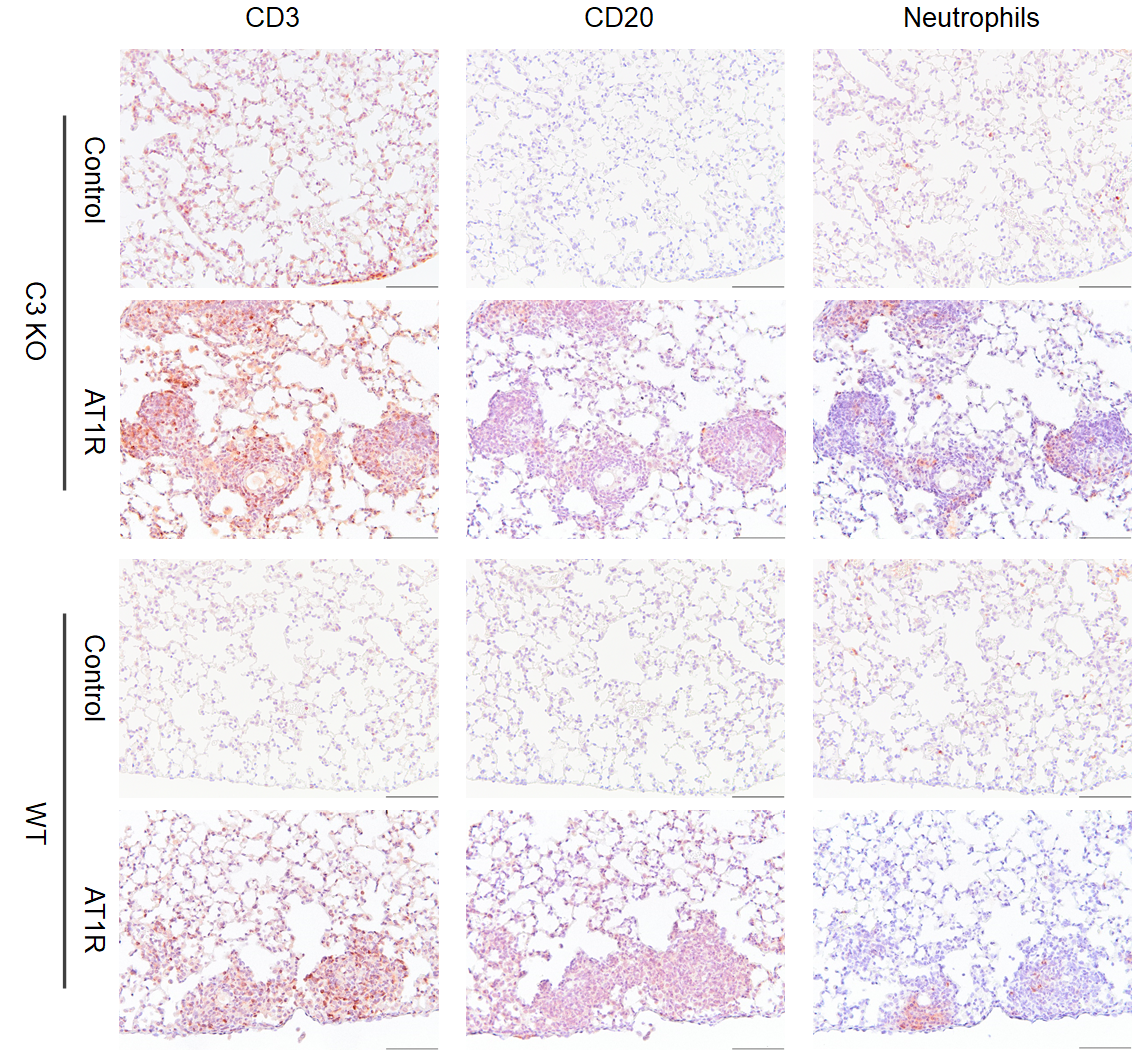


**Figure S2. Composition of inflammatory infiltrates in the lungs of mice.** Immunohistochemical staining was performed using antibodies against CD3, CD20, and neutrophils to assess the infiltration of T cells, B cells, and neutrophils, respectively, in the lung tissue. Representative micrographs from both C3-deficient and wild-type control mice immunized with hAT1R or control ME are shown. Scale bar = 100 µm.
